# Chemical Screening and Nematicidal Activity of Essential Oils from Macaronesian and Mediterranean Plants for Controlling Plant-Parasitic Nematodes

**DOI:** 10.3390/plants14030337

**Published:** 2025-01-23

**Authors:** Rui Ferreira, Carla Maleita, Luís Fonseca, Ivânia Esteves, Ivo Sousa-Ferreira, Raimundo Cabrera, Paula Castilho

**Affiliations:** 1 CQM—Madeira Chemistry Research Centre, University of Madeira, Campus da Penteada, 9020-105 Funchal, Portugal; rui.ferreira@staff.uma.pt; 2 Faculty of Exact Sciences and Engineering, University of Madeira, Campus da Penteada, 9020-105 Funchal, Portugal; ivo.ferreira@staff.uma.pt; 3 CERES—Chemical Engineering and Renewable Resources for Sustainability, Department of Chemical Engineering, University of Coimbra, Rua Sílvio Lima, 3030-790 Coimbra, Portugal; carla.maleita@uc.pt; 4 CFE-Centre for Functional Ecology—Science for People & the Planet, Associate Laboratory TERRA, Department of Life Sciences, University of Coimbra, Calçada Martim de Freitas, 3000-456 Coimbra, Portugal; luis.fonseca@uc.pt (L.F.); iesteves@uc.pt (I.E.); 5 CEAUL—Centre of Statistics and Its Applications, Faculty of Sciences, University of Lisbon, 1749-016 Lisboa, Portugal; 6 Phytopathology Unit, Biology Section, Faculty of Sciences, University of La Laguna, Avda. Astrofísico Francisco Sánchez s/n, 38204 La Laguna, Tenerife, Spain; rcabrera@ull.edu.es

**Keywords:** *Bursaphelenchus xylophilus*, chemotaxis, hatching inhibition, *Meloidogyne javanica*, mortality bioassay, *Pratylenchus penetrans*

## Abstract

Plant-parasitic nematodes are highly damaging pests responsible for heavy losses in a considerable number of plant crops. Common pest management strategies rely on the use of synthetic chemical nematicides, which have led to serious concerns regarding their impact on human health and the environment. The essential oils (EOs) obtained from aromatic plant species can provide a good source of agents for the sustainable control of nematodes, due to higher biodegradability, generally low toxicity for mammals, fish, and birds, and lower bioaccumulation in the environment. This study aimed to evaluate the nematicidal and nematostatic properties of EOs extracted from plant species relevant to Macaronesia flora or with widespread use as culinary herbs in Mediterranean cuisine. Eighteen EOs were chemically characterized and evaluated by direct contact and hatching bioassays on the root-knot nematode *Meloidogyne javanica.* The EOs that showed a significant effect on *M. javanica* second-stage juveniles’ (J2) mortality (≥40%) were also used in chemotaxis assays. From the eighteen EOs, seven showed strong nematicidal activity (>80%) and hatching inhibition. The chemotaxis assays revealed that only *Mentha pulegium* exhibited repellent behavior for *M. javanica* J2, and the rest of EOs had attractive behavior. Furthermore, EOs were assessed against the root-lesion nematode *Pratylenchus penetrans* and the pinewood nematode *Bursaphelenchus xylophilus*. *Cinnamomum burmanni* was the EO with the highest nematicidal activity for the three nematode species. Among the terpene-rich EOs, high mortality values and hatching inhibition for *M. javanica* were observed for the carvacrol chemotype *Origanum vulgare*, albeit with low activity for *P. penetrans* and *B. xylophilus*. *Mentha pulegium*, mainly composed of monoterpene ketones and monoterpenoids, demonstrated moderate-to-high mortality activity (from 30% for *P. penetrans* to 99% for *M. javanica*) for the three nematode species.

## 1. Introduction

Plant-parasitic nematodes (PPNs) are among the most widespread and harmful global pests to economically important crops and forestry and are responsible for estimated yield losses of 12.3% [1,2]. There are over 4100 described species of PPNs belonging to various families. However, numerous authors consider that this number could be underestimated, since PPN interactions with their host and consequent symptoms are often non-specific and not understood by many farmers, making it difficult to attribute crop losses to PPN damage [3,4]. Plant-parasitic nematodes display a specialized structure in the anterior region, the stylet, essential to piercing the plant cell walls and penetrating the plant tissues for feeding and reproduction [5].

PPNs can be classified according to their feeding habitats as ectoparasitic, semi-endoparasites, and endoparasites nematodes. Ectoparasites (e.g., *Belonolaimus* spp., *Dolichodorus* spp., *Longidorus* spp., *Xiphinema* spp.) maintain a vermiform anatomy throughout their life cycle and are soil dwelling, often found in the rhizosphere using roots as a transient food source and often acting as plant virus vectors [4,5]. Endoparasites can be further classified as migratory and sedentary nematodes. Root-knot nematodes (RKNs, *Meloidogyne* spp.) are an economically important group of sedentary endoparasite nematodes. The infective stage (second-stage juvenile, J2) invades the root, migrates through tissues, and establishes a permanent feeding site in the vicinity of the vascular cylinder, causing direct damage to the host and yield losses in a wide range of food crops [4,5]. RKNs induce the formation of giant cells because of repeated nuclear divisions and cortical cells proliferation, resulting in the formation of a typical gall, which is observed as a primary symptom of infection [3]. Obligate biotrophs are entirely dependent on plant-derived nutrients to fulfill their energy requirements throughout their life cycle, with a broader host range for most *Meloidogyne* species [3,5]. Another important polyphagous group of PPNs, also with an impact on economically important crops, is the migratory endoparasite the root-lesion nematode (RLN), *Pratylenchus* spp. Root-lesion nematodes can enter and leave roots during their life cycle, causing lesions, necrotic areas, browning, and cell death in infected areas, often followed by root rotting by assorted attack by fungi and/or bacteria [4,6]. The pinewood nematode, *Bursaphelenchus xylophilus*, classified as a quarantine organism by the European and Mediterranean Plant Protection Organization (EPPO) is also a migratory endoparasite nematode responsible for the pine wilt disease, a serious threat to forest ecosystems and forestry industries at a global scale [7]. The presence of this nematode obliges restrictions on the movement of plants, woody materials, and forest products, alongside implementing control and management strategies in forested areas. The life cycle and parasitism mechanism of *B. xylophilus* is distinct from other PPNs. This nematode is vectored by *Monochamus* beetles during feeding or oviposition, and the life cycle occurs inside the host tree (mainly *Pinus* spp.), including both the fungal feeding and plant feeding developmental stages [6].

Several pest management strategies are currently used against PPNs; however, no single management strategy can be considered effective. Therefore, the use of nematicides needs to be combined with other approaches, such as crop rotation and newer cultivation methodologies, pre-planting soil disinfection, the breeding of resistance crop varieties, and in the case of *B. xylophilus*, the removal, burning, or chipping of infected trees and the installation of traps to capture its insect vector [2,7,8]. Nematicides, applied to agriculture and forestry, are aimed at limiting damage to plants by reducing the number of invading nematodes or limiting the transmission of nematodes to the host. For this, a range of nematicidal products, with different active substances, modes of action, and modes of application are commercially available. However, one major concern is the non-selectivity of most common nematicides, inducing toxicity to beneficial organisms, accumulation on soil, groundwater, and foodstuffs above the maximum residue levels imposed by individual countries and organizations [9,10]. With the increase in requirements for food safety and environmental protection, highly toxic nematicides are no longer suitable for modern agriculture. Ozone-depleting nematicides, such as methyl bromide, were phased out in 2005 under the United Nations’ Montreal Protocol in developed countries [11], which was subsequently followed by the removal of most other fumigants in the market. At the same time, the use of some organophosphate and carbamate nematicides have been restricted due to their environmental implications, which has led to a further reduction in available nematicides [9,10,12]. Therefore, there is increased interest in environmentally safer control methodologies derived from natural products. Essential oils (EOs), volatile natural complex secondary metabolites obtained by mechanical extraction or hydrodistillation from aromatic plant species, are at the forefront for studies as potential use as nematicides, as their biological activities against fungi and insects are well established [13,14]. Most of the biological activities of EOs are associated with an abundance of terpene hydrocarbons and oxygenated compounds, such as alcohols and phenolic terpenes, and their degradation into nontoxic products does not appear to have any harmful effects on nontarget organisms or accumulation in the soil or groundwater [6,15,16]. There are several studies on the potential use of EOs as nematicides for *M. javanica*, *P. penetrans*, and *B. xylophilus* [6,15,17], with some constituents characterized as phenylpropanoid revealing a strong nematicidal effect.

In our study, eighteen EOs from plant species, relevant to the Macaronesia flora of Madeira (Portugal) and Canary Islands (Spain), and the chemotypes of spices and aromatic plants were extracted by hydrodistillation with a Clevenger-type apparatus. Gas chromatography coupled with flame ionizing detector (GC-FID) quantification was used for the characterization of bioactive compounds. Nematicidal/nematostatic activity was assessed against the PPNs *M. javanica*, *P. penetrans*, and *B. xylophilus*, and the toxicity thresholds and lethal concentrations causing 50% mortality (LC_50_) were calculated for the most promising EOs. Hatching activity was also studied using *M. javanica* eggs. Finally, chemotaxis assays were implemented for the evaluation of attraction/repellent behavior of the targeted EOs and main components against *M. javanica* J2.

## 2. Results and Discussion

### 2.1. EOS Phytochemical Profile

The chemical composition of each EO and the relative percentage of the major compounds are summarized in Table 1.

The quantified volatile components identified ranged from four compounds in *Argyranthemum pinnatifidum* to forty-four compounds in *Mentha pulegium*. In this study, EOs revealed a prevalence of monoterpenoids (27–92%) and monoterpenes (1–52%), except for the aromatic species *Cinnamomum burmannii*, *Ocimum gratissimum*, and *Syzygium aromaticum*, composed of phenylpropanoids (67–95%). Two of the three species of the genus *Helichrysum* (*H. devium* and *H. melaleucum*) were predominant in sesquiterpenes components.

α-phellandrene was the main terpene identified in *Apollonias barbujana* (ApSV), albeit in smaller quantities (16%) than the ones reported by Mohamed et al. [18]. Concerning *Argyranthemum pinnatifidum* (ApSV), monoterpene hydrocarbons were the most abundant, showing high proportions of β-myrcene (46%) (Table 1), also reported by Barroso et al. for this endemic species [19]; geraniol, an acyclic monoterpenoid, is also present in this EO (23%). For *Artemisia argentea* (AaPS), the monoterpene hydrocarbon α-phellandrene was the main compound (67%), followed by terpenoid camphor (12%). Figueiredo et al. reported a similar composition for this species in the only published study [20]. *Cedronella canariensis* (CcFN), a Macaronesia endemic species, had high relative amounts of pinocarvone (92%), an unusual bicyclic ketone, generally found in small amounts, with Engel et al. [21] describing a 50% pinocarvone abundance for samples collected in Tenerife and Madeira. The phenylpropanoid trans-cinnamaldehyde was the dominant EO of *Cinnamomum burmannii* (CbFX) with 91%, and no eugenol was present, as opposed to *C. verum*, regarded as “true cinnamon”, implying a positive identification as *C. burmannii* and confirming the conclusion of Wang et al. [22] and Yu et al. [23] for the common source of cinnamon in Europe and the United States.

**Figure 1:**
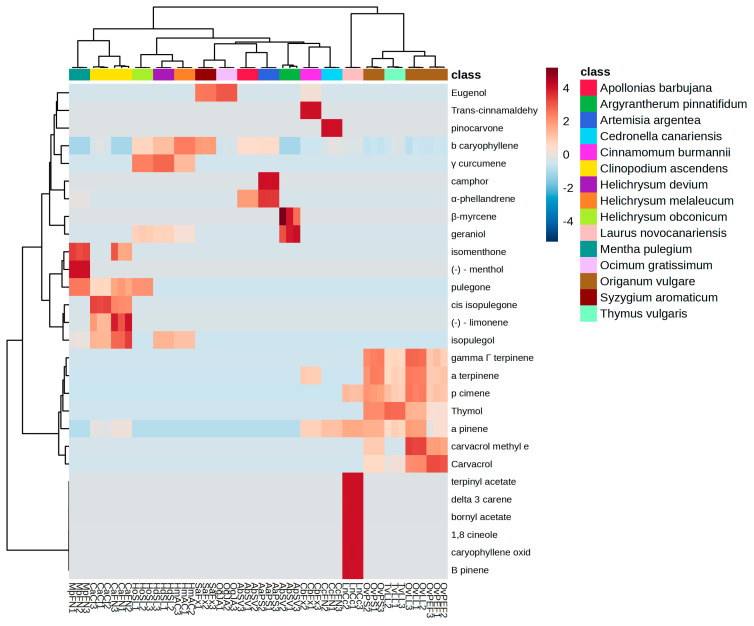
Hierarchical clustering heatmap of the essential oils profile.

EOs from *Clinopodium ascendens* had variations in terpene quantity for the two samples studied. The dominant compounds were (+)-pulegone and his isomer *cis*-isopulegone; however, the ratio of (+)-isopulegone/*cis*-pulegone content was more than double for the cultivated sample (CaCf), compared to the wild cultivar (CaFN). The relative proportion of secondary metabolites for this species is in agreement with the work of Castilho et al. [24], and variations could be attributed to the scheduled foliar fertilizing treatment and water availability for the cultivated sample. Hidalgo et al. described some differences in EO composition from samples from wild and cultivated sources, with isomenthone (37%), pulegone (17%), and 1,8-cineole (18%) as prevalent compounds [25], thus depicting the existence of different chemotypes, according to experimental data from Marongiu et al. [26]. Analyzing Table 1 and heatmap (Figure 1), the three *Helichrysum* species can be grouped into two clusters, according to their EO composition. For *H. devium* (HdSL) and *H. melaleucum* (HmAC), the bicyclic sesquiterpene (−) β-caryophyllene (15–45%) and alicyclic γ-curcumene (14–35%) were prevalent, with a reverse correlation of abundance for these metabolites between these species; for *H. obconicum* (HoSL), (+)-pulegone was more abundant (40%). Scarce information is available for these species, with most studies addressing the volatile composition for the species *Helichrysum italicum* [27,28]. Bornyl acetate represented 24% and α-terpinyl acetate about 16% for the *Laurus novocanariensis* EO (LnCc), followed by relative amounts of the hydrocarbon cyclic ether 1,8 cineole (5%), giving this sample a unique volatile profile among the studied species. These findings are notably different from that of the other species of *Laurus* (*L. nobilis* and *L. azorica*) described in the literature [29]. The EO of *Mentha pulegium* (MpFN) was rich in (+)-pulegone (54%), a monoterpene ketone, and the monocyclic monoterpenoid menthol (32%). Another study reported equivalent amounts of (+)-pulegone and (−) menthol in wild samples collected on the Chilean central coast, and the phytochemical variations are due to diverse climatic and geographical habitats [30]. For the EOs of *Ocimum gratissimum* and *Syzygium aromaticum*, the most abundant component was the phenylpropanoid eugenol (67–95%), with higher amounts in *O. gratissimum*, being grouped in the same cluster, according to Figure 1. The abundance of eugenol for both species is described in several studies [31,32,33].

A variation in the EO composition of the three samples of *Origanum vulgare* subsp. *virens* was observed. The main component of the EO obtained from a commercial source (OvPS) was the monoterpenoid thymol (59%), followed by the monoterpene biosynthetic precursor γ—terpinene (15%) and carvacrol (4%), the stereoisomer of thymol. For the sample deriving from the wild specimen (OvPEF), carvacrol was the most abundant component (73%) followed by γ—terpinene (6%) and thymol (6%). As for the sample obtained in Tenerife (OvLL), the relative proportion of the two isomers carvacrol and thymol was similar: 33% and 30%, respectively. Considering the hierarchical cluster heatmap (Figure 1), the thymol chemotype OvPS is more metabolically similar to *Thymus vulgaris* (TvLL). According to Lukas and Novak et al. [34,35], the occurrence and prevalence of secondary metabolites are consistent with the biosynthetic *p—cymyl* pathway proposed for the main compounds carvacrol/thymol, by which *γ*—terpinene is first converted to *p*—cymene and, in turn, further converted to carvacrol or thymol by two distinct hydrolases, thus establishing the main chemotypes for this species. Therefore, we can consider the presence of three chemotypes for *O. vulgare* based on the prevalence of secondary metabolites. Finally, for the EO of *Thymus vulgaris*, thymol was the prevalent compound, with a relative amount of 64%, which suggests that this EO belongs to a thymol chemotype and, thus, the results are similar to those from Borugă et al. [36] for samples cultivated in Romania. The chemical composition of the EO analyzed is, however, very different from that previously reported in Morocco and Spain for the same species of thyme [37].

### 2.2. Nematicidal Activity

#### 2.2.1. *Meloidogyne javanica*—Mortality Bioassay

**Figure 2:**
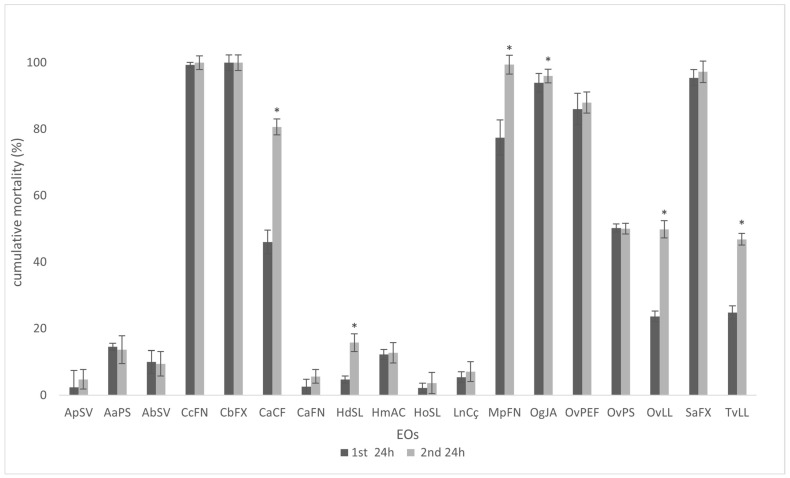
Corrected cumulative mortality (%) of *Meloidogyne javanica* second-stage juveniles, 24 h after exposure to 2000 ppm of each eighteen essential oils (grey) and 24 h after replacing the essential oils with water (blank). Water and a 0.5% EtOH with 1% Tween 20 solution were used as controls. Data are presented as an average of six triplicates, and error bars indicate the standard deviation. Significant differences (*p* < 0.05) were determined using a paired *t*-test (*) significant difference. For essential oil codes, see Table 1.

The *M. javanica* J2 mortality in 0.5% EtOH with 1% Tween 20 solution control (6.67 ± 1.75%) after 24 h of exposure was not significantly different from that observed in water control (3.94 ± 1.33%). EOs were not equally effective on *M. javanica* mortality after 24 h exposure at 2000 ppm (2.0 mg/ML), and no EO demonstrated nematostatic activity after replacing the EO with water (Figure 2). In some cases, the nematicidal activity increased, as observed for *Clinopodium ascendens* (CaCf), *Helichrysum devium* (HdSL), *Mentha pulegium* (MpFN), *Origanum vulgare* (OvLL), and *Thymus vulgaris* (TvLL), implying a lasting effect for these EOs. Among the studied EOs, *Cinnamomum burmannii* (CbFx), *Cedronella canariensis* (CcFN)*, Mentha pulegium* (MpFN)*, Ocimum gratissimum* (OgJA), and *Syzygium aromaticum* (SaFx) induced >90% mortality. Also, the carvacrol chemotype of *Origanum vulgare* subsp. *virens* OvPEF induced 86.04 ± 1.05% mortality, as opposed to 50.24 ± 5.09% for thymol-rich *Origanum vulgare* subsp. *virens* OvPS (Figure 2). The data are in agreement with the work of Oka et al. [17], who observed that EOs rich in carvacrol had a more effective nematicidal activity than those belonging to a thymol-rich chemotype. Another noteworthy result was the 23.61 ± 3.43% mortality observed after exposure to the sample acquired in “San Cristóbal de La Laguna” (OvLL) and characterized by a relative 1:1 proportion of thymol and carvacrol. These data may suggest an antagonistic behavior for the complex terpene mixture described for this sample. This hypothesis is supported by the mortality values for *Thymus vulgaris* EO (24.85 ± 0.75%), which is remarkably like the *Origanum vulgare* thymol: carvacrol chemotype (OvLL). The mortality rates for the two samples of *Clinopodium ascendens* EOs are different. Whereas the wild sample CaFN induced 2.59 ± 1.63% mortality, the cultivated sample CaCf caused 46.00 ± 2.43% of *M. javanica* J2 mortality. Again, the discrepancy can be attributed to the relative proportion of secondary metabolites, notably (+)-pulegone and *cis*-isopulegone, with a greater nematicidal impact for the latter (Table 1). This is somehow contradicted by the high activity of *Mentha pulegium* EOs, with a substantial amount of pulegone but no isopulegone; however, EOs from this species have more than 30% of menthol, which nematicidal activity is well-documented [38,39,40]. The number and position of double bonds appear to influence nematicidal activity [6].

**Figure 3:**
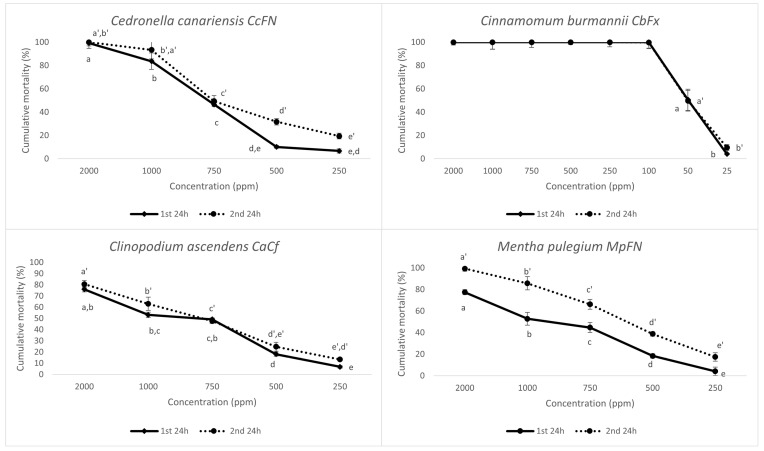

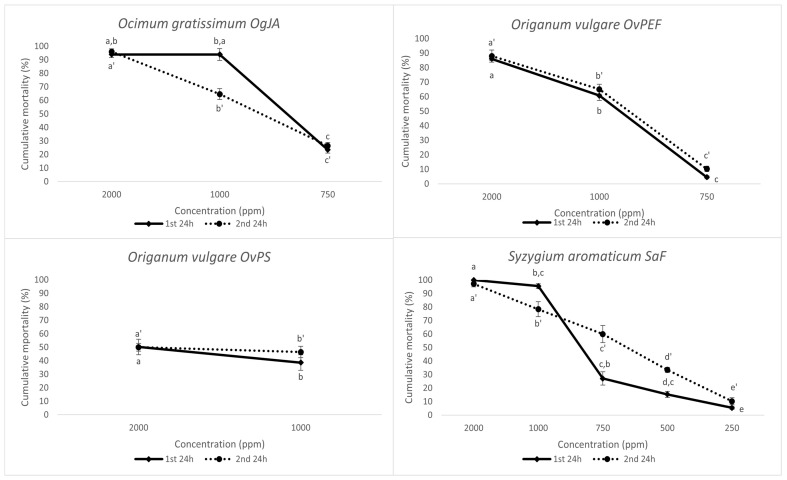
Corrected cumulative mortality (%) of *Meloidogyne javanica* second-stage juveniles, 24 h after exposure to different concentrations of the most effective essential oils (1st 24 h) and 24 h after replacing the essential oils with water (2nd 24 h). Water and a 0.5% EtOH with 1% Tween 20 solution were used as controls. Data are presented as an average of six replicates, and error bars indicate the standard deviation. Statistical analysis was performed using ANOVA followed by Tukey’s honestly significance difference (HSD) post hoc test; different letters (a, b, c, …) and (a’, b’, c’, …) represent significant differences at *p* < 0.05 for the 1st and 2nd 24 h, respectively.

A total of nine EOs with strong activity were further screened at lower EO concentrations (1000, 750, 500, 250, 100, 50, and/or 25 ppm obtained by serial dilutions) to determine toxicity thresholds and LC_50_ (Figure 3 and Table 2). A 100% mortality rate was observed 48 h after exposure to *Cinnamomum burmannii* EO for concentrations ≥ 100 ppm. As for other EOs with notable nematicidal effects, *Syzygium aromaticum*, *Mentha pulegium*, and *Cedronella canariensis* induced mortality rates ≥ 50% at 750 ppm; whereas, the EO from *Ocimum gratissimum* had a sharp decline in mortality from 64.60 ± 4.03% (1000 ppm) to 26.48 ± 2.51% (750 ppm). The samples of Origanum vulgare presented different outcomes in activity, with the carvacrol chemotype OvPEF inducing 65.14 ± 3.37% at 1000 ppm and decreasing to 10.30 ± 1.79% for 750 ppm (Figure 3). Nevertheless, this chemotype was the most effective in active *Origanum vulgare* EO.

As for LC_50_, estimated values at 24 h after exposure varied from 50.15 ppm for *Cinnamomum burmannii* to 1414.00 ppm for *Origanum vulgare* (OvPS)*,* therefore giving the former EO more nematicidal potential (Table 2).

#### 2.2.2. *Meloidogyne javanica*—Hatching Bioassay

**Figure 4:**
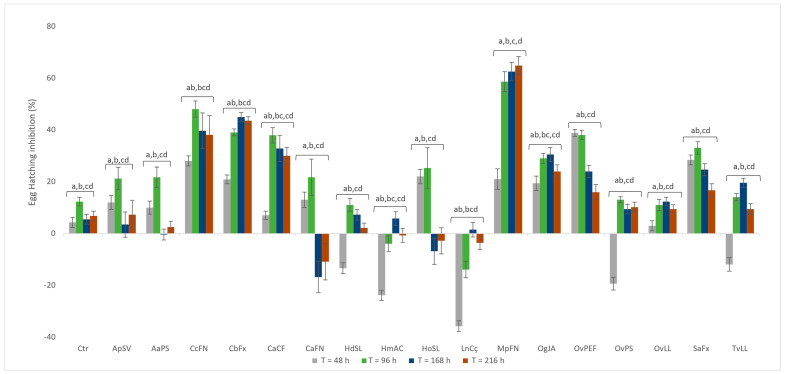
Hatching bioassay for *Meloidogyne javanica* for 216 h and exposure to 1000 ppm for each essential oil. Data are presented as an average of six replicates, and error bars indicate the standard deviation. For each EO, statistical analysis was performed using ANOVA followed by Tukey’s honestly significant difference (HSD) post hoc test. Ctr: EtOH 0.5% + Tween 20 1%; different letters represent significant differences at *p* < 0.05 at different times. For essential oil codes, see Table 1.

The results obtained for the hatching bioassay follow a trend along the previous EOs screening for J2 mortality, since most EOs that produced significant mortality in the J2 were also effective at inhibiting hatching (Figure 4). Oka et al. [17] refer to the practicality of hatching bioassays in screening EOs for nematicidal activity, because counting hatched J2 is more accurate than counting immobile or deceased juveniles in a particular J2 population. The EOs from *Clinopodium ascendens* (CaFN) and *Helichrysum obconicum* (HoSL) produced an inhibitory hatching effect at the first 96 h, with egg hatching inhibition values of 37.88 ± 3% and 25.25 ± 5%, respectively. For the rest of the duration of the assay, there was a tendency to favor egg hatching, with both Eos’ observed values above the control solvent. The result was different from that observed with *Origanum vulgare* OvPS and *Thymus vulgaris* (TvLL)*,* which have favored egg hatching for the first 48 h (19.40 ± 2% and 11.94 ± 3%, respectively) and produced an inhibitory effect for the rest of the duration of the assay (Figure 4).

*Argyranthemum pinnatifidum* (ApSV) and *Artemisia aergentea* (AaPS) EOs had similar values of hatching inhibition, starting from 12.00 ± 3% and 10.00 ± 2% at the first 48 h, peaking at 96 h with 21% inhibition for both, declining to values below 10% for the rest of the assay (Figure 4). This tendency was also observed for EOs from *Cedronella canariensis* (CcFN) and *Clinopodium ascendens* (CaCf), albeit with higher egg inhibition percentages for the last count at 216 h. This could be due to acquired resistance to EOs over time or a continuous volatilization process of terpenes present in the complex mixture of EOs. The values for the hatching inhibition observed for *Cinnamomum burmanni* EO (CbFx) are also relevant, with a verified egg inhibition percentage of 43.48 ± 2% after 216 h. Ntalli and Caboni [22] refer to high nematicidal activity against *M. javanica* for aldehydes, such as *p*-anisaldehyde, benzaldehyde, and trans-cinnamaldehyde. The EOs extracted from *Helichrysum* genus (HmAC and HoSL) and *Laurus novocanariensis* (LnCç) revealed a hatching inhibition percentage above the control solvent. Our data demonstrated an inhibition of hatching slightly above 2% for *H. devium* (HdSL). The EO extracted from *Mentha pulegium* (MpFN) was the most effective in hindering egg hatching, with inhibition values of 21.00 ± 4% for 48 h to 64.78 ± 4% after 216 h. This effect can be attributed to the abundance of pulegone and (−) menthol, as described by Abdelrasoul and El-Habashy [41] and Ntalli and Caboni [38,40,42]. *Ocimum gratissimum* (OgJA) displayed a hatching inhibition of 30.43 ± 3% at 48 h and 23.91 ± 3% at 216 h. These values are higher than that reported for the other eugenol-rich EO *Syzygium aromaticum* (SaFx) for the same timestamp and reflect the greater abundance of this phenylpropanoid in *O. gratissimum*.

As for the three chemotypes for *Origanum vulgare* subsp. *virens* (Ov), the observed inhibition percentage mimics the behavior observed for nematicidal activity, with the carvacrol chemotype (OvPEF) inhibiting 15.94 ± 3% of *M. javanica* hatching at 216 h, a higher value comparing the thymol chemotype (OvPS; 10.14 ± 2%) and carvacrol–thymol chemotype (OvLL; 9.42 ± 2%). These results are in agreement with the results previously stated in the literature [17] and also with the results obtained in our study for the nematicidal activity among each chemotype. The hatching inhibition induced by the EO from *Thymus vulgaris* (−11.94 ± 3% at 48 h, 9.42 ± 2% at 216 h) is very similar to values obtained for the thymol chemotype *Origanum vulgare* (OvPS), reflecting the abundance of thymol for the two EOs.

#### 2.2.3. *Meloidogyne javanica*—Chemotaxis Bioassay

**Figure 5:**
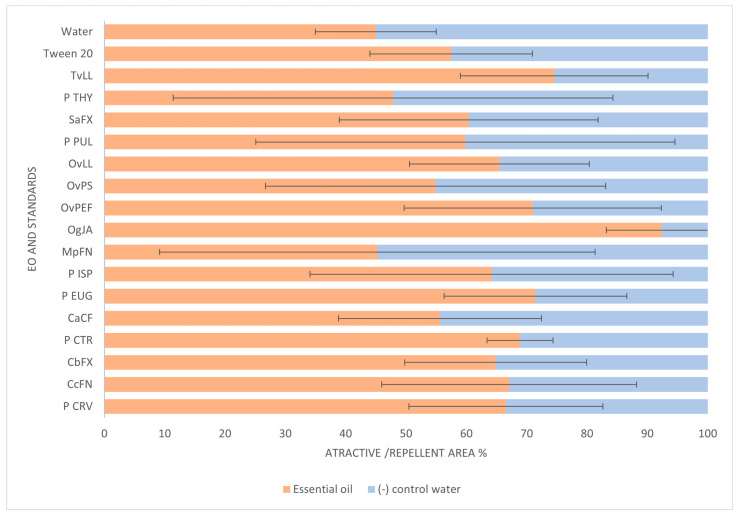
Chemotaxis bioassay for *Meloidogyne javanica* J2 with various essential oils and standards. Water and 0.5% EtOH with 1% Tween 20 solutions were included as controls. Data are presented as the average of four/five replicates, and error bars indicate the standard deviation. For essential oil codes, see Table 1. P THY: thymol CAS 89-83-8; P PUL: S (−) pulegone CAS 3391-90-0; P ISP: (−) isopulegol CAS 89-79-2; P EUG: eugenol CAS 97-53-0: P CRV: carvacrol CAS 499-75-2.

The *M. javanica* J2 chemotaxis assay results are presented in Figure 5. For the negative control Mili-Q water, both sides were selected in almost equal percentages (45.00 vs. 55.00%); however, a slight difference was observed for the control Tween 20, with *M. javanica* J2 preferring the treated area with the solvent of the EOs, compared to the Mili-Q water area (57.47 vs. 42.53%). For the tested EOs, only *Mentha pulegium* (MpFN) exhibited a repellent behavior for *M. javanica* J2, with a negative ratio between the attractive and repellent zones of −0.11, with nematodes preferring the untreated Mili-Q water area. Also, the thymol chemotype *Origanum vulgare* OvPS had an almost equal percentage between the EO and Mili-Q water control preferences (54.89 vs. 45.111%); although, the ratio of attractive–repellent zone was negative (–0.64). The rest of the EOs had attractive activities, being more evident for *Ocimum gratissimum* (OgJA; ratio of 0.85 and 92.33% of specimens in the treated area) and *Thymus vulgaris* (TvLL; ratio of 0.53 and 74.57% of specimens in the treated area). As for standards, all the tested terpenes and the phenylpropanoid eugenol revealed an attractive effect, favoring the treated area as opposed to the Mili-Q water area (Figure 5). An interesting observation refers to the effect of the monoterpenoid thymol (P THY). According to our study, *M. javanica* J2 favor the area treated with Mili-Q (52.16 vs. 47.84% for thymol area), which is opposite to the data for *Thymus vulgaris* EO (TvLL), where thymol is the prevalent compound, with 64%. The previously described effect for the thymol chemotype *O. vulgare* OvPS (59% abundance of thymol) aligns with the results for thymol. Thus, the results suggest antagonistic behavior for the complex terpene mixture identified for *Thymus vulgaris*, considering the presence and relative proportions of other terpenes, such as *p*—cymene and, especially, carvacrol, with attractive behavior in our study.

#### 2.2.4. *Pratylenchus penetrans*—Mortality Bioassay

**Figure 6:**
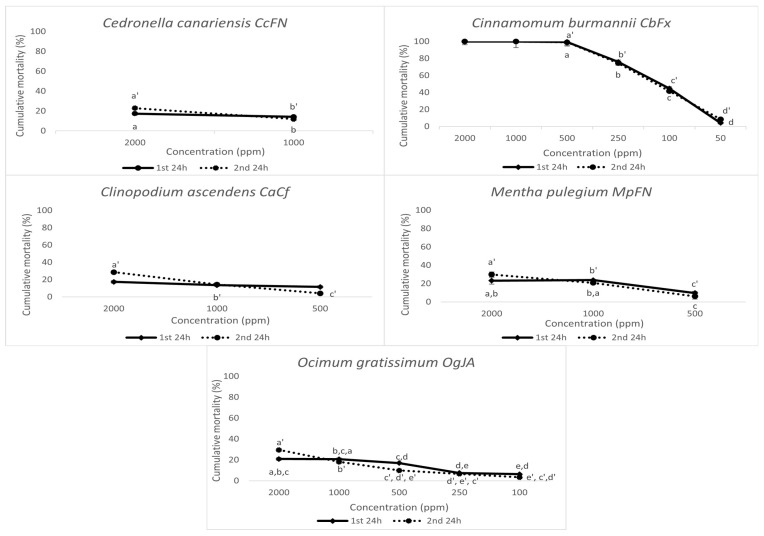
Corrected cumulative mortality (%) of *Pratylenchus penetrans* mixed developmental stages, 24 h after exposure to different concentrations of the most effective essential oils (1st 24h) and 24 h after replacing the essential oils with water (2nd 24 h). Water and a 0.5% EtOH with 1% Tween 20 solution were used as controls. Data are presented as an average of six replicates, and error bars indicate the standard deviation. Statistical analysis was performed using ANOVA followed by Tukey’s honestly significant difference (HSD) post hoc test; different letters (a, b, c, …) and (a’, b’, c’, …) represent significant differences at *p* < 0.05 for the 1st and 2nd 24 h, respectively.

*P. penetrans* mortality with the EO solvent (0.5% EtOH with 1% Tween 20) was 16.61 ± 2.3% at the highest concentration of 2000 ppm, which is not significantly different from the mortality values observed in water (10.53 ± 1.5%). Of the seven EOs that showed nematicidal activity >70% at 2000 ppm for *M. javanica*, the EO from *Cinnamomum burmanni* (CbFx) presented a 100% mortality rate to concentrations ≥500 ppm, being the most effective EO (Figure 6). The abundance of trans-cinnamaldehyde, as the main compound in *C. burmannii*, may be responsible for this outcome. This observation is validated by Barbosa et al. [43], who state that compounds resulting from the phenylpropanoid pathway have a significant nematicidal activity to *P. penetrans*. *Clinopodium ascendens* (CaCf) and *Mentha pulegium* (MpFN) had a mortality rate >25% at 2000 ppm, even after replacing the EO with water and a second 24 h incubation period, thus reflecting the moderately induced mortality by (+) pulegone, isopulegone, and (−) menthol. However, the nematode mortality with *C. ascendens* EO decreased to 13.54 ± 0.82% at 1000 ppm, rendering this EO less effective, and a nematostatic effect was observed at a concentration of 500 ppm, since the mortality decreased after the removal of the EO. For the *Cedronella canariensis* EO (CcFN), the mortality at 2000 ppm was 22.95 ± 1.26% after the second 24 h incubation period, decreasing to 11.94 ± 1.47% at a concentration of 1000 ppm. The *Ocimum gratissimum* (OgJA—29.55 ± 0.98%) and *Syzigium aromaticum* (SaFx—25.33 ± 3.78%) EOs had similar mortality rates >25%, which can be attributed to considerable amounts of eugenol present, as shown in Table 1 (67–95%). Contrary to the observation from Barbosa et al. [43], the carvacrol-rich EO *Origanum vulgare* OvPEF only induced 16.75 ± 2.73% at 2000 ppm, as opposed to complete mortality described by that author.

Analyzing LC_50_ values, the *Cinnamomum burmanni* EO had the highest nematicidal effect on *P. penetrans*, with 99.38 ppm for the first 24 h incubation and 100.20 ppm after the EO removal and a further 24 h incubation in water. This LC_50_ was significantly higher than that calculated for this EO with *M. javanica* J2 (50.15 ppm for the first 24 h, 51.29 for the second 24 h). This tendency is further noticeable when comparing LC_50_ values for *M. javanica* J2 (Table 1) and *P. penetrans* mixed development stages (Table 3), which confirms a lower sensitivity for *Pratylenchus* to some nematicides compared to *Meloidogyne,* reported in the literature [44]. However, four EOs stood out for their high LC_50_ values, all with theoretical values surpassing 5000 ppm: *Clinopodium ascendens* (CaCf), *Cedronella Canariensis* (CcFN), *Mentha pulegium* (MpFN), and *Ocimum Gratissimum* (OgJA).

#### 2.2.5. *Bursaphelenchus xylophilus*—Mortality Bioassay

**Figure 7:**
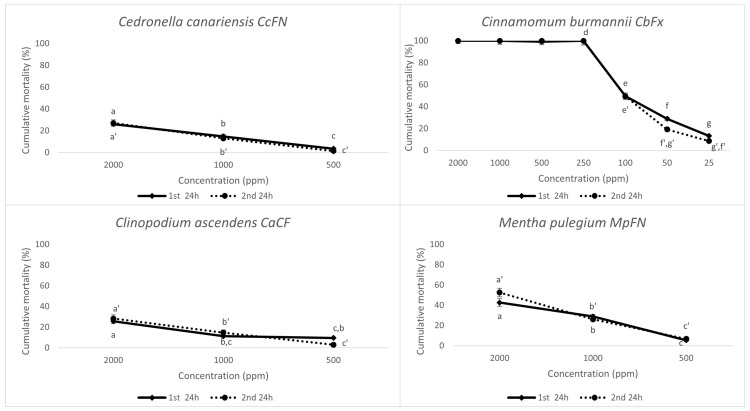
Corrected cumulative mortality (%) of *Bursaphelenchus xylophilus* mixed developmental stages, 24 h after exposure to different concentrations of the most effective essential oils (−) and 24 h after replacing the essential oils with water. Water and a 0.5% EtOH with 1% Tween 20 solution were used as controls. Data are presented as the average of six replicates, and error bars indicate the standard deviation. Statistical analysis was performed using ANOVA followed by Tukey’s honestly significant difference (HSD) post hoc test; different letters (a, b, c, …) and (a’, b’, c’, …) represent significant differences at *p* < 0.05 for the 1st and 2nd 24 h, respectively.

As seen for the other two nematode species, the 24 h mortality rate for the EO solvent at the highest concentration of 2000 ppm (10.67 ± 1.26%) was not significantly different from that observed in water (8.37 ± 1.03%). EOs showing nematicidal activity ≥ 40% at 2000 ppm were screened at lower concentrations, as described (Figure 7).

As observed in the *M. javanica* and *P. penetrans* mortality bioassay, *Cinnamomum burmannii* (CbFx) induced 100% nematode mortality for concentrations ≥ 250 ppm (Figure 7). The other noteworthy EO, *Mentha pulegium* (MpFN), induced a mortality rate of 42.65 ± 3.74% at 2000 ppm after 24 h incubation and increased to 52.60 ± 4.23% after the removal of the EO solution and replacement with water. The *Cedronella canariensis* EO (CcFN) revealed a moderate nematicidal activity for PWN, with a mortality rate of 26.10 ± 2.56% at 2000 ppm for the first 24 h and 27.05 ± 2.93% at the second 24 h incubation period. This tendency was also observed in the *P. penetrans* mortality bioassay. The results suggest low activity of the main compound pinocarvone for PWN and RLN. It is noteworthy to compare the weaker nematicidal activity of *Clinopodium ascendens* (CaCf) in comparison with *Mentha pulegium* (MpFN). Both EOs have (+)-pulegone in significant concentrations (54% for *M. pulegium*, 32% for *C. ascendens*), but the presence of monoterpenoid (−) menthol (32%) in *M. pulegium* may produce a synergistic interaction between the two compounds that enhances the nematicidal activity [2,45]. Interestingly, the eugenol-rich *Ocimum gratissimum* (OgJA) and *Syzygium aromaticum* (SaFx) EOs that had induced > 90% mortality in *M. javanica* J2 (Figure 2) show the weakest activity for PWN within the tested EOs, with 9.47 ± 4.43% and 7.45 ± 0.63 mortality at 2000 ppm (2 mg/ML) in the first 24 h, respectively, in the same conditions. These results reflect the higher tolerance of *B. xylophilus* to phytochemicals and other nematicides, with one study referring to eugenol as one of the highest LC_50_ for this pathogen [6]. The data for the carvacrol chemotype *Origanum vulgare* (OvPEF) disclose a weak-to-moderate anti-PWN activity (15.49 ± 2.94% mortality rate for the first 24 h and 17.45 ± 3.19% for the second 24 h incubation period). These results are in sharp contrast to the ones observed for *Mentha pulegium* (MpFN), reaffirming the conclusion for substantial tolerance of *B. xylophilus* for the majority of the tested EOs. Various authors refer to the role of structural characteristics, such as the types of functional groups, saturation, and carbon skeleton in determining the toxicity of EOs and other plant metabolites for *B. xylophilus* [2,6,46]. Another study by Barbosa et al. [47] concerning the biological activity screening for fifty-two EOs against *B. xylophilus* revealed that thirteen EOs were highly effective, resulting in more than 90% mortality at 2000 ppm (2 mg/mL), with six of them resulting in 100% mortality. LC_100_ values ranged between 500 ppm (0.50 mg/mL) and 830 ppm (0.83 mg/mL) for the EOs of *Origanum vulgare* and *Satureja montana*, respectively. Faria et al. [2] evaluated the toxicity of eight EOs against *B. xylophilus* by comparing 1:1 EOs mixtures, to assess for potential synergistic interactions. The mixtures of *Cymbopogon citratus–Mentha piperita* EOs and of *Foeniculum vulgare*–*Satureja montana* showed the highest activities among single EOs and mixtures, with LC_50_ of 0.09 and 0.05 µL/mL for the latter. Based on the second 24 h incubation in water, after a 24 h EO incubation, the *Cinnamonum burmannii* EO (CbFx) was the most toxic towards *B. xylophilus* with a LC50 value of 106.60 ppm, followed by *Mentha pulegium* (MpFN; 1959.10 ppm) (Table 4). The LC50 values of *Clinopodium ascendens* (CaCf) and *Cedronella canariensis* (CcFN) were in the same threshold, with theoretical values above 7000 ppm (Table 4).

## 3. Materials and Methods

### 3.1. Chemicals and Standards

Standards used for identification purposes with GC-FID were as follows: (−) pulegone (98%); (−)-isopulegone (99%); (+)-p-menth-1-ene (98%); (−)-citronellal (98%); ƴ- terpinene (99%); α-terpinene (95%); p-cymene (99%); (−)-α- pinene (98%); (−)-β-pinene (99%); R (+)-limonene (97%); and eugenol (99%) (Fluka™, Seelze, Germany). Thymol (99%); carvacrol (98%); and carvacrol methyl ether (98%) were acquired from Merck KGaA (Darmstadt, Germany). n-hexane was acquired from PanReac AppliChem (Barcelona, Spain). For nematicidal/nematostatic activity, ethanol (96%) and sodium hypochlorite were acquired from Carlo Erba™ (Chau. du Vexin, France) and Tween 20 from PanReac AppliChem™ (Barcelona, Spain).

### 3.2. Sample Preparation

Fresh leaves or stems from different species important for the Macaronesia flora (*Apollonias barbujana*, *Argyranthemum pinnatifidum*, *Artemisia argentea*, *Cedronella canariensis*, *Laurus novocanariensis*, and three species of *Helichrysum* (*H. devium*, *H. melaleucum*, and *H. obconicum*)) or with widespread use as culinary herbs in Mediterranean cuisine (*Cinnamomum burmannii*, *Mentha pulegium*, *Ocimum gratissimum*, *Origanum vulgare*, *Syzygium aromaticum*, and *Thymus vulgaris*) were obtained (Table 1). Plant parts from *O. vulgare* subsp. *virens* were obtained from three diverse geographical sources: “Parque Ecológico do Funchal”, Madeira Island (wild specimen); “Ponta do Sol”, Madeira Island (cultivated); and “San Cristóbal de La Laguna”, Tenerife, Spain (cultivated) (Table 1). Also, two samples of Clinopodium ascendens were obtained from different Madeira origins: “Fajã da Nogueira” (wild specimen) and “Centro de Fruticultura” (cultivated by micropropagation from a wild specimen) (Table 1). Vouchers for endemic species were deposited in the Madeira Botanical Garden Eng. Rui Vieira. Leaves were dried for 48 h using a ventilated food dehydrator at 40 °C.

### 3.3. Essential Oil Isolation

The dried plant material was ground into a fine powder using a mechanical grinder to achieve particle sizes <250 µm. EOs were obtained by hydrodistillation for 4 h at a ratio of 1:20 (*w*/*v*) using a Clevenger-type apparatus. The EOs were stored in an opaque flask at 4 °C.

### 3.4. Gas Chromatography–Flame Ionization Detector (GC-FID) Conditions

The EOs were prepared by adding 1 mL of n-hexane to 20 mg of each EO and analyzed using a GC-FID method. The EOs characterization was performed using an Agilent 7890A gas chromatography (Agilent, Santa Clara, CA, USA) equipped with an autosampler Agilent 7693, a SPB™FA fused silica capillary column (30 m × 0.25 mm × 0.2 µm), and an FID detector. Helium was used as the carrier gas at a flow rate of 800 µL/min. The GC oven temperature started at 60 °C for 2 min, increased to 220 °C at 2 °C/min, and held for 20 min, with a total runtime of 102 min. The injector and FID detector temperatures were held at 250 °C, respectively. One microliter of EO–hexane mixture was injected at a split ratio of 60:1, with a delay time of 4 min. Air and hydrogen were supplied to the FID detector at flow rates of 400 and 40 mL/min, respectively. The chromatographical analysis was performed using the proprietary software A.01.04. All assays were carried out in triplicate to guarantee statistical reliability. A cluster heatmap was generated by MetaboAnalyst 6.0 software (Alberta, CA, USA).

The validation method was assessed in terms of selectivity, linearity, and sensitivity. Linearity was determined by raging the individual concentration and by plotting the relative area versus the concentration. The limit of detection (LOD) and limit of quantification (LOQ) were calculated by multiplying the standard deviation of the calibration curve’s intercept by 3 and 10, respectively, and dividing each by the slope of the corresponding linear regression equation.

### 3.5. Nematode Isolates

Three PPN species were obtained from the NEMATO-lab culture collection (CFE, UC, Portugal): *M. javanica*, *P. penetrans*, and *B. xylophilus*.

The RKN *M. javanica* isolate (PtJ) was originally obtained from the infected roots of potatoes (Guarda, Portugal) and kept on tomato cv. Coração-de-Boi pots, with sterilized sandy loam soil, sand, and substrate (1:1:1 *v*/*v*) [48]. Pots were kept in a growth chamber, at 25 ± 2 °C and 12:12 h light: dark. Concerning the mortality bioassay, egg masses were handpicked from the infected roots of tomatoes and placed in a hatching chamber. Hatched J2 from the first 24 h were discarded and subsequent J2, from the second 24 h, were collected and stored at 4 °C, until a maximum of 5 days. Before the assay, J2 were washed with sterile tap water using a 20 µm sieve, and a nematode suspension was obtained by rinsing the sieve with sterile tap water. For the hatching bioassay, egg masses were collected from infected roots, and eggs were extracted using a 0.5% sodium hypochlorite (NaOCl) solution under agitation for three minutes [49].

The RLN *P. penetrans* isolate (A44L4) was obtained from the infected roots of potatoes (Coimbra, Portugal) and was reared in vitro on carrot discs, following the protocol of Castillo et al. [1]. A nematode suspension consisting of mixed developmental stages was obtained from carrot discs by rinsing discs through a 20 µm sieve with sterile tap water.

The PWN *B. xylophilus* isolate (BxPT17AS) was collected from maritime pine, *Pinus pinaster*, at Alcácer do Sal, Portugal, and kept in cultures of *Botrytis cinerea* grown on malt extract agar medium at 25 °C. Mixed developmental nematode stages were collected from fungal cultures by washing the plate through a 20 µm sieve [50].

### 3.6. Nematicidal/Nematostatic Activity Bioassay

For each nematode species, direct-contact bioassays were performed in flat-bottom 96-well microtiter plates (Carl Roth GmbH & Co. KG, Karlsruhe, Germany). The EOs were solubilized in a solution of 0.5% EtOH with 1% Tween 20 under agitation to obtain a final concentration of 4000 ppm (4.0 mg/mL). A given volume of an aqueous suspension of nematodes, including 40–60 specimens, was added to each well, and the same volume of EO was added to obtain a final concentration of 2000 ppm (2.0 mg/mL). Water and the solution of 0.5% EtOH with 1% Tween 20 were included as controls, to assess natural nematode mortality and the effect of the organic solvent, respectively. Due to their inherent high toxicity, no nematicides were used as positive control, since the objective of this study was to evaluate the nematicidal/nematostatic potential of the EOs through a screening in vitro assay, as observed in other published studies employing this methodology [51,52,53,54]. Instead, positive controls were introduced in subsequent stages, such as pot or field trials, to assess the effectiveness of formulations or products when applied to soil environments.

The microtiter plates were sealed with plastic film to prevent EO volatilization and kept for 24 h under darkness at 25 ± 1 °C. To determine the nematicidal/nematostatic activity of each EO, after 24 h, live and immotile specimens were counted under a stereomicroscope. Then, the EO solution was removed and replaced by sterilized tap water to confirm, after another 24 h, the nematicidal or nematostatic activity of the EOs. Each treatment consisted of six replicates. EOs that showed an effect ≥ 70% on *M. javanica* mortality at 2000 ppm were screened at 1000 ppm (1.0 mg/mL) and for lower concentrations (750 (0.75 mg/mL), 500 (0.5 mg/mL), 250 (0.25 mg/mL), 100 (0.1 mg/mL), 50 (0.05 mg/mL), and 25 ppm (0.025 mg/mL)), obtained by serial dilutions, to determine toxicity thresholds, whenever an effect > 50% on mortality was observed.

Since *P. penetrans* and *B. xylophilus* are less sensitive to conventional fumigant nematicides compared to *M. javanica*, mostly due to their migratory endoparasitism and absence of permanent feeding sites [42,43,55], only the EOs that showed ≥70% effect on *M. javanica* mortality were further evaluated against *P. penetrans* and *B. xylophilus.*

### 3.7. Hatching Bioassay

The hatching bioassays were performed with *M. javanica*, in flat-bottom 96-well microtiter plates, following the same procedure described for mortality bioassay, with some adaptations. A total of 50 eggs, in approximately 50 µL of nematode suspension, were transferred into each well and the same volume of each 2000 ppm EO solution was added to obtain a final concentration of 1000 ppm. The plates were also sealed and maintained in the same conditions referred to above. Nematode hatching was checked at 2-day intervals for 9 days. Each treatment consisted of six replicates.

### 3.8. Chemotaxis Bioassay

In order to analyze whether the targeted EOs attract, repel, or have no effect on *M. javanica* J2 behavior, a combined and modified version of the methodology described by Hewlett et al. [56], Zhai et al. [57], and Petrikovszki et al. [58] was applied. Chemotaxis was studied on 1% water agar in 5.5 cm diameter Petri dish (7 mL/Petri dish). Circular wells (0.5 cm Ø) were performed on opposite sides of each Petri dish, and 50 µL of EO or Mili-Q water was pipetted into the wells. Approximately, 20 J2 (±10 µL) were placed at the center of each plate, and the plates maintained in the dark at 24 °C. After 24 h, the number of J2 in the attractive and repellent zones was recorded, according to Petrikovszki et al. [58]. Treatments were replicated 4/5 times, and the experiment was repeated twice. The results were presented as a percentage of the ratio between attractive and repellent zones. Only the EOs that showed a significant effect on *M. javanica* J2 mortality (approximately ≥ 40%) were used in the chemotaxis assay at the lower concentration with the highest effect. The main component of each EO was also tested at the same concentration of the EO: carvacrol, (−)-citronellal, eugenol, (−)-isopulegol, (−) pulegone, and thymol.

### 3.9. Statistical Analysis

All assays were carried out in triplicates to guarantee statistical significance. The data were analyzed by one-way analysis of variance (ANOVA) followed by Tukey’s honestly significant difference (HSD) post hoc multiple comparison test using IBM SPSS Statistic 29.0.1.0 software (IBM Corp, Armonk, NY, USA). Data on mortality were converted to percentage cumulative mortality and corrected by the Schneider–Orelli formula [59], concerning experimental solvent control.

(1) $$\%\;cumulative\;mortality=\left(\frac{{\%\;mortality}_{EO\;treatment}-{\%\;mortality}_{control}}{100-{\%\;mortality\;}_{control}}\;\right)\times 100$$

The mortality data were logarithmically transformed, and the assumptions of normality and homogeneity of variances were assessed with the Shapiro–Wilk and Levene’s tests, respectively, before conducting ANOVA. Results were considered statistically significant at 95% confidence level (*p* < 0.05). Data on mortality and hatching, derived from 24 and 48 h observations for mortality and 9 days for hatching, were also subjected to Probit analysis [60] using GraphPad Prism 10 software (GraphPad Software, Boston, MA, USA), and the lethal concentrations causing 50% mortality (LC_50_) calculated.

The percentage inhibition of hatching for each EO concentration was calculated using the modified formula with reference to experimental solvent control [61,62].

(2) $$\%\;Hatching\;inhibition=\left(\frac{Hatched\;J2\;in\;control-Hatched\;J2\;in\;treatment\;}{Hatched\;J2\;in\;control}\right)\times 100$$

## 4. Conclusions

The present work evaluated the nematotoxicity of 18 EOs, extracted from distinct species relevant to the Macaronesian flora or commonly used as culinary herbs in Mediterranean cuisine. The selected EOs showed antifungal and insect antifeeding activities in previous studies (Rui Ferreira, unpublished result). To our knowledge, nine plant species (*Apollonias barbusana*, *Argyranthemum pinnatifidum*, *Artemisia argentea*, *Cedronella canariensis*, *Clinopodium ascendens*, *Helichrysum devium*, *H. melaleucum*, *H. obconicum*, *and Laurus novocanariensis*) were evaluated for the first time for their nematicidal potency against PPNs. Seven EOs caused an effect on *M. javanica* J2 mortality (>80%) and hatching at 2000 ppm, being further tested at lower concentrations and evaluated for attraction/repellent behavior with a methodology of area choice. Nematicidal activity from these seven EOs were further assessed in *P. penetrans* and *B. xylophilus*.

The EO of *Cinnamomum burmanni*, with trans-cinnamaldehyde as a major constituent, displayed high mortality values and lower LC50 values for the three studied PPN species, attaining full mortality at concentrations > 100 ppm in the case of *M. javanica*, 500 ppm for *P. penetrans*, and 250 ppm for *B. xylophilus*. These data corroborate the findings described in previous studies about the potential use of EOs from *Cinnamomum* plants, as broad-spectrum nematicides [39,46]. However, in the chemotaxis assay, this EO revealed an attractive behavior, with nematodes favoring the treated area, suggesting an attractive behavior at higher concentrations.

EOs rich in eugenol, such as those from *Ocimum gratissimum* and *Syzygium aromaticum*, induced > 90% mortality in *M. javanica* but low activity for *P. penetrans* and *B. xylophilus*, with mortality rates < 30%, while also showing an attractive response. This highlights a substantial tolerance of *B. xylophilus* to phenylpropanoid eugenol, as suggested by Sarri et al. [39].

Among the terpene-rich EOs, high mortality values and hatching inhibition for *M. javanica* were observed with phenol monoterpenes, particularly carvacrol but only moderately for the isomer thymol. This observation is validated by the distinct effect for the three chemotypes of *Origanum vulgare* (OvPEF, OvPS and OvLL). The carvacrol chemotype (OvPEF) was more effective than the thymol (OvPS) or carvacrol: thymol (OvLL) chemotypes, indicating that quantitative variations in EO monoterpene composition and isomerism appear to influence activity against RKNs, as theorized by Oka et al. [17]. However, the carvacrol chemotype demonstrated lower mortality against *P. penetrans* and *B. xylophilus*.

The EO from *Mentha pulegium* (MpFN), containing primarily (+)-pulegone and (−) menthol, display strong nematicidal activity against *M. javanica* but moderate effects against *P. penetrans* and *B. xylophilus*. As for attractive/repellent behavior, *Mentha pulegium* EO was classified as repellent for *M. javanica* J2.

The lowest *M. javanica* mortality activity was observed with EOs composed of mono-, sesquiterpenes and acetate ester monoterpenoid fractions, such as (−) β caryophyllene, γ-curcumene, bornyl acetate, and α-phellandrene, which were present in the EOs of *Helichrysum* species, *Laurus novocanariensis*, and *Artemisa aergentea*.

In conclusion, the essential oils (EOs) assessed in this study demonstrate varying levels of nematotoxic activity, with *Cinnamomum burmanni* and *Mentha pulegium* showing notable promise for inclusion in sustainable pest management strategies. To successfully integrate these EOs into pest control systems, further research is required to elucidate the mechanisms of action of their primary constituents. Additionally, research on potential synergistic or antagonistic interactions, alongside assessments of the impact on nontarget organisms are essential for optimizing their efficacy. Future studies should also prioritize the development of “user-friendly” formulations, addressing aspects such as controlled release, environmental degradation, and cost-effectiveness.

## Figures and Tables

**Table 1:** Essential oils included in this study: code, origin, ID voucher, extraction yield, and profile characterization by gas chromatography with flame ionizing detector.

Plant Species	Code	Origin	ID Voucher	Yield (*v*/*w*, %)	Major Compounds ^1^
*Apollonias barbujana*	AbSV	São Vicente—Madeira	MADJ104321	0.05	α-phellandrene (16.01 ± 0.13); (−) β-caryophyllene (6.09 ± 0.08)
*Argyrantherum pinnatifidum*	ApSV	São Vicente—Madeira	MADJ14543	0.11	β -myrcene (45.91 ± 0.28); geraniol (23.33 ± 0.04)
*Artemisia argentea*	AaPS	Porto Santo—Madeira	MADJ15231	0.19	α-phellandrene (66.83 ± 0.162); camphor (12.44 ± 0.08); (−) β-caryophyllene (6.34 ± 0.09)
*Cedronella canariensis*	CcFN	Fajã da Nogueira—Madeira	MADJ24356	1.10	pinocarvone (92.40 ± 1.67); (−) β-caryophyllene (2.27 ± 0.59)
*Cinnamomum burmannii*	CbFx	Market Funchal—Madeira	n.a.^1^	0.62	trans cinnamaldehyde (90.46 ± 3.97)
*Clinopodium ascendens*	CaCf	Centro de Fruticultura—Madeira	n.a.^2^	1.37	cis—isopulegone (70.17 ± 1.93); (+)-pulegone (21.26 ± 0.40); isopulegol (1.50 ± 0.44)
	CaFN	Fajã da Nogueira—Madeira	MADJ306406	1.42	cis- isopulegone (48.71 ± 1.75); (+)-pulegone (32.34 ± 1.70); isopulegol (13.99 ± 0.25)
*Helichrysum devium*	HdSL	Ponta de São Lourenço—Madeira	MADJ210758	0.44	γ-curcumene (34.73 ± 1.06); (−) β-caryophyllene (15.35 ± 0.06)
*H. melaleucum*	HmAC	Achadas da Cruz—Madeira	MADJ210803	0.32	(−) β-caryophyllene (45.12 ± 0.26); γ-curcumene (13.67 ± 0.98)
*H. obconicum*	HoSL	Ponta de São Lourenço—Madeira	MADJ210810	0.04	(+)-pulegone (40.03 ± 0.45); γ-curcumene (28.00 ± 0.16)
*Laurus novocanariensis*	LnCç	Caniço—Madeira	n.a.^2^	0.36	bornyl acetate (23.72 ± 0.02); α terpinyl acetate (15.64 ± 0.04); 1,8-cineole (5.02 ± 0.03)
*Mentha pulegium*	MpFN	Fajã da Nogueira—Madeira	MADJ302861	0.67	(+)-pulegone (54.26 ± 0.29); (−) menthol (31.90 ± 0.02); (+) isomenthone (2.08 ± 0.12)
*Ocimum gratissimum*	OgJA	Jardim das Aromáticas—Madeira	n.a.^1^	0.30	eugenol (94.56 ± 1.02); (−) β-caryophyllene (1.10 ± 0.26)
*Origanum vulgare subsp. virens*	OvPEF	Parque Ecológico do Funchal—Madeira	MADJ306206	1.90	carvacrol (73.04 ± 1.07); γ-terpinene (5.97 ± 0.38); thymol (5.65 ± 0.47)
	OvPS	Ponta do Sol—Madeira	n.a.^1^	2.01	thymol (59.19 ± 0.90); γ-terpinene (14.81 ± 0.75); carvacrol (4.16 ± 0.35)
	OvLL	San Cristóbal de La Laguna—Tenerife, Canary Island	n.a.^1^	2.04	carvacrol (32.18 ± 1.54); thymol (30.91 ± 0.60); γ-terpinene (18.77 ± 0.75)
*Syzygium aromaticum*	SaFx	Market Funchal—Madeira	n.a.^1^	1.00	eugenol (67.48 ± 1.18); (−) β-caryophyllene (29.68 ± 0.95)
*Thymus vulgaris*	TvLL	San Cristóbal de La Laguna—Tenerife, Canary Island	n.a.^1^	0.37	thymol (63.79 ± 0.56); p-cymene (16.00 ± 0.67); carvacrol (6.91 ± 1.36)

^1^ Relative percentage (% ± standard deviation); mean of three replicates. n.a.^1^: commercially acquired; n.a.^2^: micropropagated from a wild specimen.

**Table 2:** Estimated values of 50% lethal concentration (ppm) of *Meloidogyne javanica* second-stage juveniles’ mortality 24 h after exposure (1st 24 h) to eight essential oils and 24 h after replacing the essential oils with water (2nd 24 h).

Essential Oil	Code	Lethal Concentration (LC_50_, ppm)		Gradient Equation R^2^	
		1st 24 h	2nd 24 h	1st 24 h	2nd 24 h
*Cedronella canariensis*	CcFN	782.30 (758.0 ± 807.10)	818.40 (n.a. ± 858.90)	y = −25.874x + 126.91 R^2^ = 0.9511	y = −22.264x + 125.62 R^2^ = 0.9429
*Cinnamomum burmannii*	CbFx	50.15 (48.64 ± 51.77)	51.29 (50.06 ± 53.41)	y = −10.937x + 131.05 R^2^ = 0.5578	y = −10.527x + 129.77 R^2^ = 0.5671
*Clinopodium ascendens*	CaCf	576.90 (541.60 ± 617.90)	766.00 (702.00 ± 840.10)	y = −17.348x + 92.713 R^2^ = 0.9604	y = −17.281x + 97.745 R^2^ = 0.9927
*Mentha pulegium*	MpFN	714.50 (660.60 ± 770.50)	684.30 (619.30 ± 748.20)	y = −18.111x + 93.835 R^2^ = 0.9812	y = −21.083x + 124.77 R^2^ = 0.9873
*Ocimum gratissimum*	OgJA	950.30 (n.a.)	889.30 (n.a.)	y = −35.237x + 140.93 R^2^ = 0.7500	y = −34.742x + 131.83 R^2^ = 0.9968
*Origanum vulgare* subsp. *virens*	OvPEF	845.20 (n.a.)	840.40 (n.a.)	y = −40.712x + 131.90 R^2^ = 0.9541	y = −38.856x + 132.19 R^2^ = 0.9466
	OvPS	1408.00 (n.a.)	1414.00 (n.a.)	y = −11.570x + 61.808 R^2^ = 1	y = −3.556x + 53.596 R^2^ = 1
*Syzygium aromaticum*	SaFx	714.60 (654.40 ± 779.90)	677.80 (610.30 ± 743.50)	y = −26,927x + 129.46 R^2^ = 0.8770	y = −21,892x + 121.53 R^2^ = 0.9945

**Table 3:** Estimated values of 50% lethal concentration (ppm) of *Pratylenchus penetrans* mixed developmental stages 24 h after exposure (1st 24 h) to five essential oils and 24 h after replacing the essential oils with water (2nd 24 h).

Essential Oil	Code	Lethal Concentration (LC_50_, ppm)		Gradient Equation R^2^	
		1st 24 h	2nd 24 h	1st 24 h	2nd 24 h
*Cedronella canariensis*	CcFN	8361.00 (estim.)	5965.00 (estim.)	y = −3.2291x + 20.573 R^2^ = 1	y = −11.014x + 33.965 R^2^ = 1
*Cinnamomum burmannii*	CbFx	99.38 (53.61 ± 129.00)	100.20 (53.99 ± 152.30)	y = −19.101x + 137.56 R^2^ = 0.8323	y = −18.783x + 136.36 R^2^ = 0.8507
*Clinopodium ascendens*	CaCf	4580.00 (estim.)	5813.00 (estim.)	y = −2.9089x + 19,998 R^2^ = 0.9653	y = −12.263x + 40.204 R^2^ = 0.9906
*Mentha pulegium*	MpFN	4003.00 (estim.)	5904.00 (estim.)	y = −6.7306x + 32.355 R^2^ = 0.7137	y = −11.923x + 42.847 R^2^ = 0.9826
*Ocimum gratissimum*	OgJA	6102.00 (estim.)	3663.00 (estim.)	y = −4.2339x + 27.163 R^2^ = 0.9238	y = −6.3945x + 32.704 R^2^ = 0.9238

**Table 4:** Estimated values of 50% lethal concentration (ppm) of *Bursaphelenchus xylophilus* mixed developmental stages, 24 h after exposure (1st 24 h) to four essential oils and 24 h after replacing the essential oils with water (2nd 24 h).

Essential Oil	Code	Lethal Concentration (LC_50_, ppm)		Gradient Equation R^2^	
		1st 24 h	2nd 24 h	1st 24 h	2nd 24 h
*Cedronella canariensis*	CcFN	7883.00 (estim.)	7575.00 (estim.)	y = −11.253x + 37.343 R^2^ = 1	y = −12.833x + 39.472 R^2^ = 0.9969
*Cinnamomum burmannii*	CbFx	110.90 (98.53 ± 122.90)	106.60 (98.53 ± 115.00)	y = −16.132x + 134.68 R^2^ = 0.8220	y = −17.360x + 137.63 R^2^ = 0.8161
*Clinopodium ascendens*	CaCf	9875.00 (estim.)	7762.00 (estim.)	y = −8.0721x + 31.716 R^2^ = 0.8241	y = −12.661x + 40.684 R^2^ = 0.9985
*Mentha pulegium*	MpFN	2313.00 (estim.)	1959.10 (estim.)	y = −18.634x + 62.918 R^2^ = 0.9774	y = −22.924x + 74.332 R^2^ = 0.9919

## Data Availability

The original contributions presented in this study are included in the article. Further inquiries can be directed to the corresponding author.
